# TSG-6 Downregulates IFN-Alpha and TNF-Alpha Expression by Suppressing IRF7 Phosphorylation in Human Plasmacytoid Dendritic Cells

**DOI:** 10.1155/2017/7462945

**Published:** 2017-03-06

**Authors:** L. Kui, G. C. Chan, P. P. W. Lee

**Affiliations:** Department of Paediatrics and Adolescent Medicine, LKS Faculty of Medicine, The University of Hong Kong, Pokfulam, Hong Kong

## Abstract

Proinflammatory cytokines such as TNF-*α* and type I interferons (IFN) are pathogenic signatures of systemic lupus erythematosus, and plasmacytoid dendritic cells (pDCs) play a major role by predominantly producing IFN-*α*. Given the rise of importance in identifying tumor necrosis stimulated gene 6 (TSG-6) as a key anti-inflammatory regulator, we investigate its function and its ability to counteract proinflammatory cytokine secretion by pDCs in vitro. CpG-A and R837 induced significant endogenous TSG-6 expression in the pDC cell-line GEN2.2. Following recombinant human TSG-6 treatment and CpG-A or R837 stimulation, significant reduction in IFN-*α* and TNF-*α* was observed in healthy donors' pDCs, and the same phenomenon was confirmed in GEN2.2. By CD44 blocking assay, we deduced that the suppressive effect of TSG-6 is mediated by CD44, by reducing IRF-7 phosphorylation. Our findings suggest that TSG-6 and its downstream signalling pathway could potentially be targeted to modulate proinflammatory cytokine expression in pDCs.

## 1. Introductions

Interferons and proinflammatory cytokines are key players in regulating and directing the innate and adaptive immunity of our body against intracellular pathogens and viruses. However, overexpression of these cytokines and interferons can be harmful with pathological outcome. Type I interferons (IFN), such as interferon-alpha (IFN-*α*), and proinflammatory cytokines such as tumor necrosis factor-alpha (TNF-*α*) play an important role in the pathogenesis and disease progression of systemic lupus erythematosus (SLE) [1]. Thus, appropriately regulating the expression of type I IFN and proinflammatory cytokines is pivotal in the control of autoimmune diseases.

Dendritic cells (DCs) are immune cells that bridge the link between the innate and adaptive immunity [2]. Amongst the DCs subsets, plasmacytoid dendritic cells (pDCs) are known to be professional type I IFNs producing cells as they express high levels of both TLR7 and TLR9 intracellular receptors. These receptors are capable of recognising ssRNA and unmethylated CpG-containing DNA ligands [3, 4], with a constitutively high IRF7 expression [5], which can readily mount an immediate antiviral response. IRF7 is a key transcriptional factor involved in regulating IFN-*α* gene in lymphoid cells [6]. Phosphorylation of IRF7 is necessary for IFN-*α* signal transduction, followed by nuclear translocation and activation of type I IFN gene transcription [7, 8]. However, when the expression of type I IFN is dysregulated, adverse immunological effects may result. Notably, pDCs found in the peripheral blood and lymph nodes are the major source of IFN-*α*, and they also express proinflammatory cytokines like TNF-*α* [9], upon stimulation via TLR7/9 receptors [10].

Therefore, elucidating the factors and regulatory mechanisms of IFN and TNF-*α* will not only enable us to understand the role of pDCs in host defense against foreign antigens but also allow us to understand the feedback mechanism in maintaining immune homeostasis, preventing autoimmunity. TNF-stimulated gene 6 (TSG-6) is one of such factors that exerts anti-inflammatory effects as suggested by several groups [11–21]. This immune suppressive effect is mainly operated by regulating chemokine functions [22], participating in the negative feedback mechanism of inflammatory machinery [23].

Using differential screening of cDNA library prepared from TNF-stimulated human diploid FS-4 fibroblasts, Wisniewski and colleagues [24] reported that TNF-*α* and IL-1 could induce TSG-6 expression. Recent studies also showed TSG-6 as a potential biomarker for human mesenchymal stem cell (hMSC) with immunosuppressive properties in treating sterile inflammation [25]. Other evidences supporting the potential therapeutic effect of TSG-6 include (1) anti-inflammatory and wound healing effect in acute transplant rejection setting [26, 27]; (2) delaying onset of autoimmune diabetes [28]; (3) improving liver regeneration [29]; and (4) suppressing inflammation after traumatic brain injury in mice [30].

Hydroxychloroquine, an antimalarial drug, has been demonstrated to be an effective treatment for SLE patients, as it acts by downregulating proinflammatory cytokines as well as IFN-*α* in these patients [31, 32]. Similarly, TSG-6 has also been observed by Choi and colleagues [23] to have an anti-inflammatory role in regulating the inflammatory cytokines expression in the TLR/NF-kB pathway, via the CD44 receptor. This provides evidences that TSG-6 may play a role in regulating type I IFN and proinflammatory cytokines. Further understanding of the immunomodulatory effects of TSG-6 would be important in adopting it as an adjunct therapy for autoimmune disorders.

In this study, we used human pDC cell-line GEN2.2 and healthy donor PBMCs stimulated with human TLR-9 specific agonist CpG-A ODN2216, which is a potent IFN-*α* inducer, and TLR-7 specific agonist R837 (Imiquimod). We analysed how TLR7/9-activated pDCs responded to TSG-6 and showed that proinflammatory cytokine and type I IFN such as TNF-*α* and IFN-*α* could be downregulated by TSG-6. Moreover, CpG-A and R837 could readily induce TSG-6 expression and CD44 transcription. We also showed that this effect was mediated by CD44 and downregulation of IRF-7 phosphorylation.

## 2. Materials and Methods

### 2.1. Preparation of PBMCs

Peripheral blood mononuclear cells (PBMCs) were isolated from buffy coat preparations of healthy voluntary blood donors, by Ficoll-Hypaque gradient centrifugation. Cells were washed in phosphate buffered saline (PBS) and suspended in RPMI 1640 medium supplemented with penicillin, streptomycin, and 10% heat-inactivated Fetal Bovine Serum (FBS).

### 2.2. pDC Cell-Line GEN2.2

The pDC Cell-line Gen2.2 (kindly provided by Dr. Joel Plumas and Dr. Laurence Chaperot, Research and Development Laboratory, French Blood Bank Rhône-Alpes, Grenoble, France) require murine stromal cells (MS5) as feeder cells to propagate (Chaperot et al., 2006). MS5 were obtained from DSMZ (Braunschweig, Germany), irradiated at 60 Grays, and cultured with Gen2.2 grown in complete medium (RPMI 1640 Glutamax; GibcoBRL) supplemented with 1 mM sodium pyruvate, penicillin, streptomycin, nonessential amino acids, and 10% heat-inactivated Fetal Bovine Serum (FBS).

### 2.3. TLR Stimulation

PBMC (1 × 10^7^) and GEN2.2 (1 × 10^6^) were cultured with CpG-A ODN2216 (2 *μ*M; InvivoGen, San Diego, CA, USA) or R837 (10 ng/mL; Imiquimod, InvivoGen, San Diego, CA, USA) and media, pretreated with recombinant human TSG-6 (rhTSG-6) (1000 ng/mL; R&D Systems cat #2104-TS-050) overnight, at 37°C in 5% CO_2_. The duration of CpG-A or R837 stimulation was 3 hours for RT-qPCR, 6 hours for TNF-*α* and IFN-*α* quantification by ELISA, 30 minutes for pIRF7 expression by flow-cytometric analysis, and 5 hours for phenotypic and intracellular cytokine analysis by flow cytometry. Brefeldin A (GolgiPlug, BD Pharmingen, San Diego, CA, USA) was added during the final two hours of stimulation to block cytokine secretion, prior to intracellular cytokine staining for flow analysis.

### 2.4. Effects of Blocking Antibody to CD44 in Human pDC Cell-Line GEN2.2

GEN2.2 was plated at 0.5 × 10^6^ cells/cm^2^ in 24-well microplates in 500 *μ*L GEN2.2 complete medium. The samples were incubated for 45 min with 5 *μ*g/mL, 10 *μ*g/mL, and 20 *μ*g/mL of human CD44 blocking antibody, clone BRIC 235 (NHS Blood and Transplant, University of Bristol), or 5 *μ*g/mL of mouse isotype control (IgG_2b_, InvivoGen mabg2b-ctrlm) and then incubated with TSG-6 (1000 ng/mL) overnight, followed by stimulation with CpG-A or R837 for 6 hours.

### 2.5. ELISA

Supernatants were collected from cell cultures following treatment with TSG-6 and stimulation with respective stimulants CpG-A or R837 for 6 hours, for analysis by ELISA (Human TNF-alpha DuoSet ELISA kit, R&D Systems; Human TSG-6 ELISA kit, RayBio; Human IFN-*α* ELISA kit, ThermoFischer Scientific).

### 2.6. Flow Cytometry

The panels of antibodies used for phenotypic and intracellular cytokine or IRF7 and CD44 detection are described in detail under Supplementary Table S1 (in Supplementary Material available online at https://doi.org/10.1155/2017/7462945).

Cytokines IRF7 and CD44 detection and phenotyping were performed by sequential cell surface or/and intracellular staining, following the manufacturer's instructions. Fluorescence activated cell sorting (FACS) analysis was performed on a three-laser BD LSR-II flow cytometer, and data were analysed using FlowJo Software v8.1 (Treestar, Ashland, OR, USA) and transferred into analysis and graphic software GraphPad Prism5 (La Jolla, CA, USA). Gating strategy used for identifying the human pDCs subset from the total PBMC is shown in Supplementary Figure 2.

### 2.7. RNA Isolation and Quantitative Real-Time PCR

Total RNA was extracted from GEN2.2 in TRIzol reagent (Invitrogen Life Technologies). Reverse transcription to cDNA was carried out by methods using RevertAid RT Kit (ThermoScientific). These cDNA was used for real-time PCR by an ABI-Prism 7900HT PCR machine (Applied Biosystems). The list of primer sequence can be found in supplementary Table S2.

### 2.8. Statistical Analysis

Data were expressed as mean value of ± SEM from at least three independent experiments and analysed by Student's *t*-test, One-Way ANOVA, Fisher's Least Significant Difference (LSD) test, and Wilcoxon matched-pairs signed rank test, with GraphPad Prism 6.01 software.

## 3. Results

### 3.1. TSG-6 Reduced Both TLR-7 and TLR-9 Mediated TNF-*α* and IFN-*α* Expression in Human pDCs Cell-Line GEN2.2

TSG-6 has been shown by Choi and colleagues [23] to have an anti-inflammatory role in regulating inflammatory cytokines expression via the TLR/NF-kB pathway in murine resident macrophages. Therefore, we would like to see if similar findings can be observed in human pDCs. In addition, we would like to check whether pDCs could regulate not only TNF-*α*, but also IFN-*α* expression via TLR7 and TLR9 pathways.

**Figure 1:**
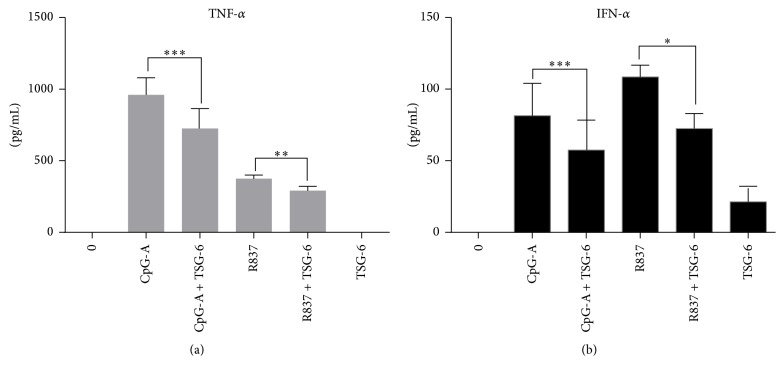
TSG-6 decreased TLR7/9 mediated TNF-*α* and IFN-*α* production in GEN2.2 cells. ELISA analysis of TNF-*α* (a) and IFN-*α* (b) in GEN2.2 cells treated with TSG-6 (1000 ng/mL) overnight, with or without stimulation with CpG-A at 2 *μ*M or R837 at 10 ng/mL for 6 hours. Graphs show data from three independent experiments performed in duplicate. Error bars indicate SEM. Paired Student's *t*-test, ^*∗*^*p* < 0.05, ^*∗∗*^*p* < 0.01, and ^*∗∗∗*^*p* < 0.001.

First, we stimulated GEN2.2 using CpG-A and R837 and showed that rhTSG-6 could decrease the protein expression of TNF-*α* and IFN-*α* in both TLR9 (Figure 1(a)) and TLR7 (Figure 1(b)) mediated pathways. A dose response of GEN2.2 pretreated with different concentrations of rhTSG-6 (10–1000 ng/mL) was performed to optimize the concentration used for subsequent experiments (Fig S1). In these and subsequent experiments, cells were pretreated with rhTSG-6 at 1000 ng/mL overnight.

**Figure 2:**
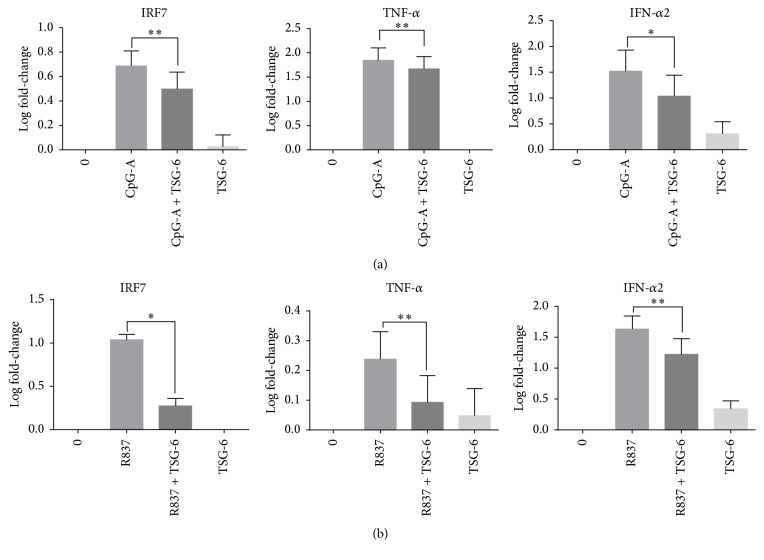
TSG-6 reduced the transcription of interferon regulatory factor 7 and its associated genes in GEN2.2. TSG-6 reduced the transcription of interferon regulatory factor 7 and its associated genes in GEN2.2. Real-time PCR analysis of IRF7, IFN-*α*2, TNF-*α*, and IL-1*β* in GEN2.2 cells treated with TSG-6 (1000 ng/mL) overnight, followed by stimulation with CpG-A at 2 *μ*M (a) or R837 at 10 ng/mL (b) for 3 hours. Graphs show data of more than 3 experiments performed in duplicate. Error bars indicate SEM. One-Way ANOVA, ^*∗*^*p* < 0.05, ^*∗∗*^*p* < 0.01.

Since IRF7 is a key transcription factor of both TLR7 and TLR9 mediated type I IFN expression, it would be interesting to know if rhTSG-6 would also affect the transcriptional level of IRF7 and its target genes. As expected, we showed that rhTSG-6 downregulated IRF7, IFN-*α*2, and TNF-*α* via both TLR9 (Figure 2(a)) and TLR7 (Figure 2(b)) mediated pathways in GEN2.2 after 3 hours of CpG-A or R837 stimulation. A more significant decrease was seen in TLR7 mediated IFN-*α*2 and TNF-*α* transcription (*p* < 0.01). The TSG-6 only treatment on pDCs did not upregulate nor downregulate IRF7, TNF-*α*, or IFN- *α*.

These data showed that TSG-6 potentially modulates and suppresses both TNF-*α* and IFN-*α* in TLR7/9 activated pDCs at the transcriptional level as well as the protein level.

### 3.2. Phosphorylation of IRF7 Was Reduced by TSG-6 in Human pDCs Cell-Line GEN2.2

**Figure 3:**
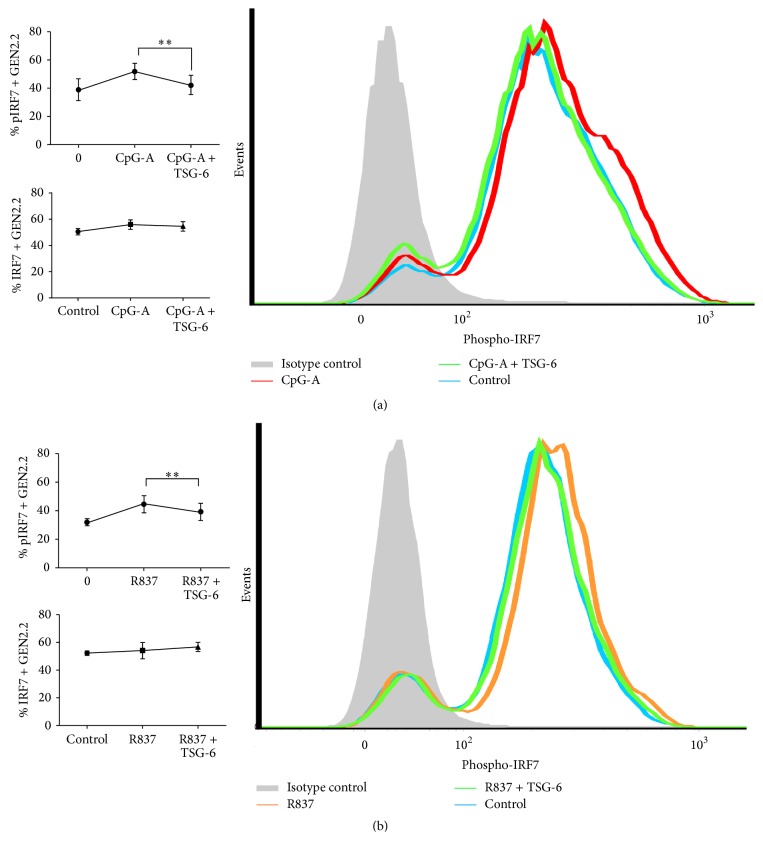
TSG-6 reduced the activation of IRF7 in GEN2.2 cells. GEN2.2 cells were treated with TSG-6 (1000 ng/mL) overnight, followed by stimulation with CpG-A (2 *μ*M) (a) or R837 (10 ng/mL) (b) for 30 min. The cells were then fixed, permeabilized, and stained to detect phosphorylated IRF7 (phosphor-IRF7) at Ser477 and Ser479 by flow cytometry. The bar chart shows the means of 5 independent experiments, indicating the IRF7 phosphorylation activity in GEN2.2 cells. Error bars indicate SEM. Paired Student's *t*-test, ^*∗∗*^*p* < 0.01.

As IRF-7 is a multifunctional transcription factor, tight regulation by posttranslational modification, such as phosphorylation, is important and indicative of its activation. As we observed that the mRNA level of IRF-7 was reduced upon rhTSG-6 treatment in GEN2.2 within 3 hours of stimulation by CpG-A and R837 (Figures 2(a) and 2(b)), we next investigated whether IRF-7 activation could also be affected. Using intracellular staining of Ser477/Ser479 phospho-IRF7 and analysis by flow cytometry, we found that rhTSG-6 reduced the phosphorylation of IRF-7 in GEN2.2 stimulated by CpG-A (Figure 3(a)) or R837 (Figure 3(b)) for 30 minutes, while total IRF-7 expression (PE-expressing population) remained unchanged.

Taken together, our findings revealed that TSG-6 reduced phosphorylation of IRF-7 and resulted in the downregulation of IRF-7, TNF-*α*, and IFN-*α* expression.

### 3.3. Expression of TSG-6 Was Induced by TLR7/9 Agonists CpG-A and R837 in GEN2.2

**Figure 4:**
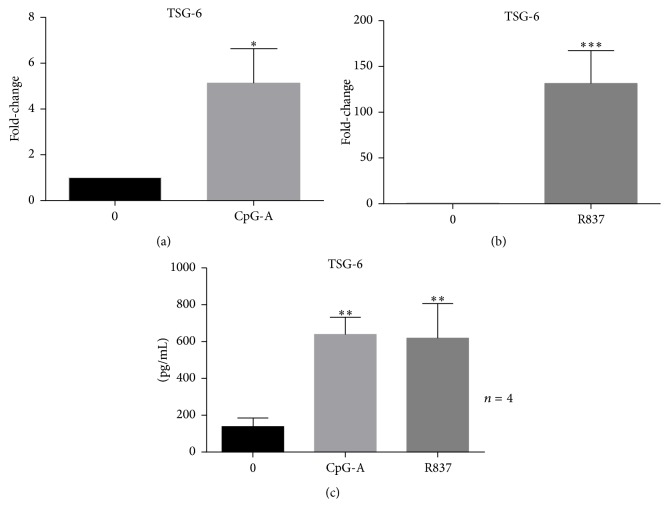
CpG-A and R837 induced TSG-6 expression in GEN2.2 cells. (a and b) Real-time PCR analysis and (c) ELISA analysis of GEN2.2 cells treated with CpG-A (2 *μ*M) or R837 (10 ng/mL) shows induction of TSG-6 at both transcription and protein level. Graphs show data of at least 3 independent experiments performed in duplicate. Error bars indicate SEM. Paired Student's *t*-test, ^*∗*^*p* < 0.05, ^*∗∗*^*p* < 0.01, and ^*∗∗∗*^*p* < 0.001.

Since our previous experiments suggested that TSG-6 might exert an immune-regulatory effect on human pDCs, we next evaluated whether CpG-A and R837 would exert an effect on TSG-6 expression. We observed that both CpG-A and R837 stimulation would induce TSG-6 expression in GEN2.2. CpG-A and R837 not only induced mRNA transcription of TSG-6 at 3 hours after stimulation (Figures 4(a) and 4(b)) but also significantly increased TSG-6 protein expression (Figure 4(c)) at 6 hours after stimulation (*p* < 0.01). These results revealed that the immune suppressive protein, TSG-6, may be an important molecule upregulated by the activation of TLR9 and TLR7 signalling pathways, in response to CpG-A and R837 stimulation, respectively.

### 3.4. TSG-6 Increased Expression of CD44 on Human pDCs Cell-Line GEN2.2

Recent studies have realized the importance of CD44, the cell surface receptor in exerting a negative regulatory effect on TLR-2 signalling in macrophages [33], and that the inhibitory effects of TSG-6 on macrophages were dependent on CD44 [23]. Thus, in our following experiments, we looked into the importance of CD44 in our study of TSG-6 regulation in the TLR7/9 pathway, by using two strategies.

**Figure 5:**
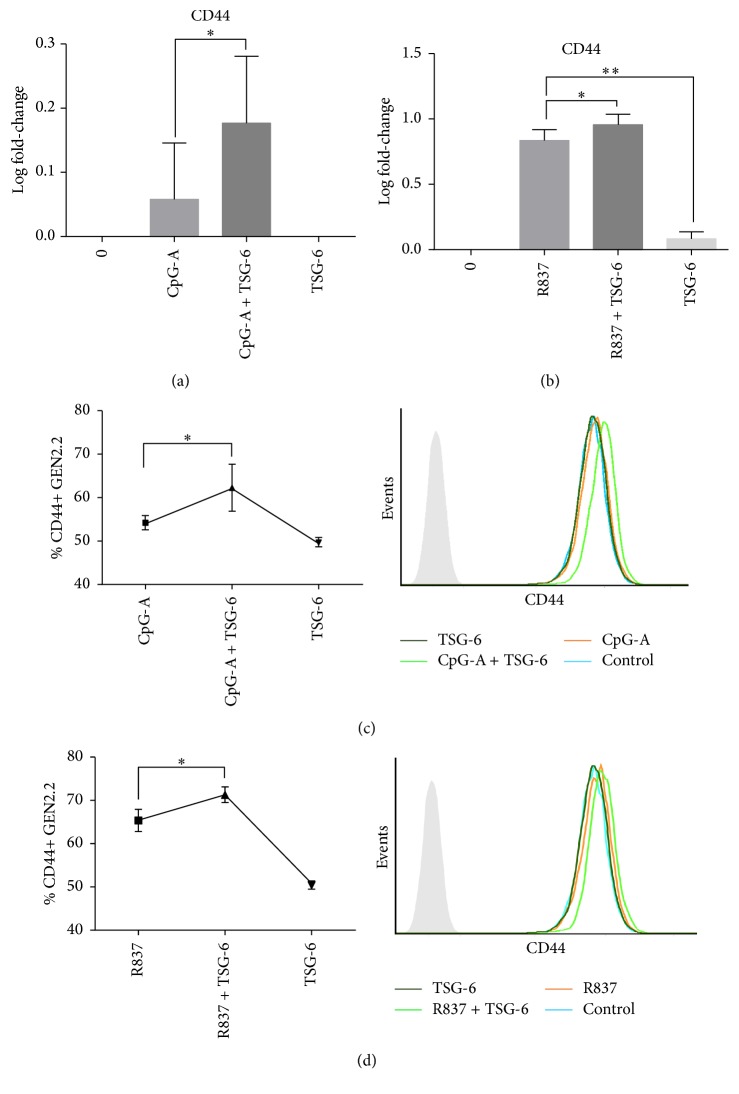
TSG-6 increased CD44 expression. Real-time PCR (a and b) and flow-cytometric analysis (c and d) of GEN2.2 cells treated with TSG-6 (1000 ng/mL), followed by stimulation with CpG-A (2 *μ*M) or R837 (10 ng/mL) for 3 hours. Representative FACS plots showing CD44+ pDCs percentages per independent experiment (*n* > 3). Graphs showing data of at least 3 experiments performed in duplicate. Error bars indicate SEM. One-Way ANOVA, ^*∗*^*p* < 0.05, ^*∗∗*^*p* < 0.01.

Firstly, we attempted to see if exogenous rhTSG-6 treatment added to TLR9/TLR7 stimulated pDC would enhance CD44 expression. We found that CpG-A (Figure 5(a)) and R837 (Figure 5(b)) stimulated GEN2.2 treated with rhTSG-6 could both increase the transcriptional level of CD44 as well as cell surface expression of CD44 (Figures 5(c) and 5(d)).

**Figure 6:**
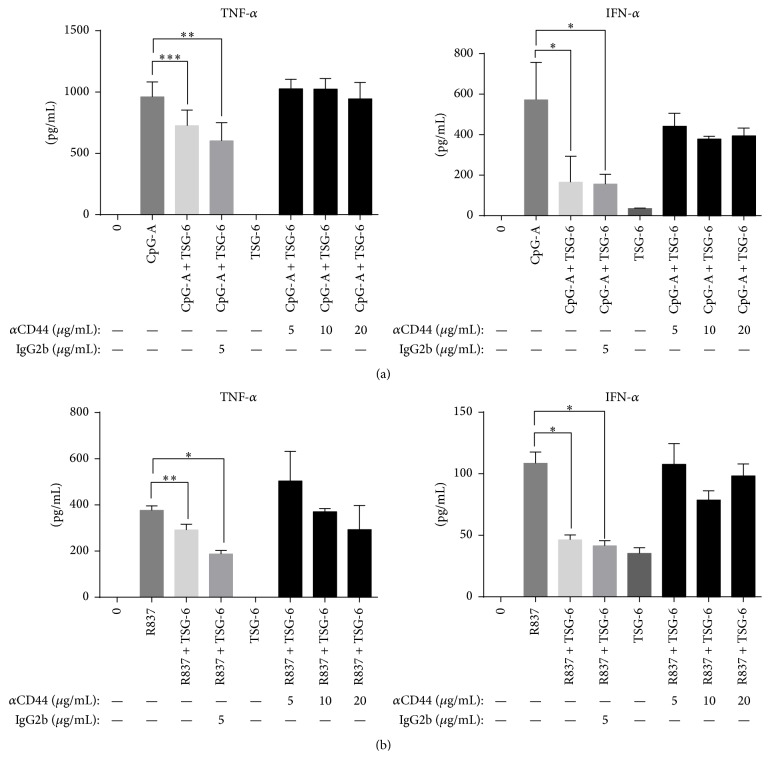
The suppressive effect of TSG-6 was dependent on CD44. GEN 2.2 cells were treated with anti-CD44 blocking antibody at various concentrations as indicated or with control IgG (mouse IgG2b) for 45 min, followed by TSG-6 (1000 ng/mL) overnight, and stimulation with CpG-A (2 *μ*M) (a) or R837 (10 ng/mL) (b) for 6 hours. Culture supernatants were collected for TNF-*α* and IFN-*α* quantification by ELISA. Graphs showing data from three independent experiments performed in duplicate. Error bars indicate SEM. One-Way ANOVA, ^*∗*^*p* < 0.05, ^*∗∗*^*p* < 0.01, and ^*∗∗∗*^*p* < 0.001.

Then we used an anti-CD44 blocking antibody BRIC 235 to block the interaction of hyaluronan with CD44 [34]. This could further delineate the role of CD44 in mediating the immunosuppressive action of TSG-6. Blocking of CD44 receptors on human pDCs cell-line GEN2.2 using BRIC 235 would eliminate the suppressive effect of rhTSG-6 in both CpG-A (Figure 6(a)) and R837 (Figure 6(b)) stimulated GEN2.2. Both TNF-*α* and IFN-*α* protein expression levels in these experiments were similar when compared with cells stimulated with CpG-A or R837 alone.

Taken together, not only did we observe an induction of CD44 expression by TSG-6 in human pDCs stimulated with CpG-A or R837, but more importantly, the regulatory effect of TSG-6 seen in the human pDCs was mediated by CD44.

### 3.5. Suppressive Effect of TSG-6 on Human Peripheral Blood pDCs Stimulated with CpG-A or R837

**Figure 7:**
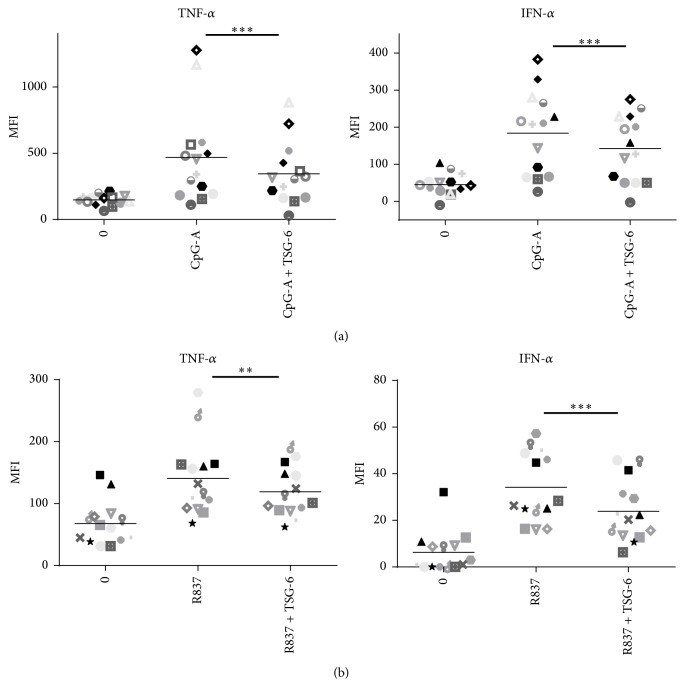
TSG-6 exerted the same suppressive effect on pDCs from healthy donors stimulated with CpG-A or R837. PBMC from 14 healthy blood donors were isolated and treated with TSG-6 (1000 ng/mL) overnight, followed by CpG-A (2 *μ*M) (a) or R837 (10 ng/mL) (b) for 5 hours. The cells were stained with the cell surface markers as indicated, fixed, and permeabilized, followed by staining with APC-conjugated anti-IFN-*α* and FITC-conjugated anti-TNF-*α* antibodies. PBMCs were gated for pDCs as defined in the gating strategy (Supplementary data S2). MFI, mean fluorescence intensity. Error bars indicate SEM. Wilcoxon matched-pairs signed rank test was used to determine statistical significance. ^*∗∗*^*p* < 0.01, ^*∗∗∗*^*p* < 0.001.

Since we observed the immune-regulatory role of TSG-6 in GEN 2.2, we would like to see if the same phenomenon could also be observed in pDCs in peripheral blood from healthy donors. For this experiment, a similar experimental condition (rhTSG-6 1000 ng/mL pretreatment overnight, followed by CpG-A or R837 stimulation for 5 hours, and BFA for 2 hours) was employed. The pDCs population (HLA-DR+, Lineage1−, CD11c−, CD123+) was gated from PBMC (according to the gating strategy shown in Supplemental Fig.2), and intracellular TNF-*α* and IFN-*α* were analysed by flow cytometry. Indeed, the same phenomenon was observed in pDCs gated from the PBMC of 14 healthy donors. Both TNF-*α* and IFN-*α* in CpG-A (Figure 7(a)) or R837 (Figure 7(b)) stimulated pDCs were downregulated significantly when they were pretreated with TSG-6. TSG-6 pretreatment alone did not increase nor decrease the intracellular level of TNF-*α* and IFN-*α* (data not shown). We showed that TSG-6 treatment alone did not significantly affect pDCs. Taking into consideration that the cells were preincubated with TSG-6 prior to CpG-A or R837 stimulation, this result did not reflect the influence of TSG-6 on other cell-types, nor its systemic effect during in vivo situation.

In summary, the data from the experiments done on both GEN2.2 and healthy donors' PBMC suggested that the immunoregulatory effects of TSG-6 on TLR7 and TLR9 pathways were conducted by downregulating TNF-*α* and IFN-*α* expression via decreasing IRF-7 phosphorylation, and such an effect was dependent on CD44.

## 4. Discussion

Our results unfold a novel mechanism in which rhTSG-6 abated the surge of inflammatory cytokine TNF-*α* and IFN-*α* produced by human pDCs when stimulated with CpG-A or R837. These data not only extend our knowledge of the suppressive effect of TSG-6 mediated by downregulation of IRF-7 phosphorylation via TLR7 or TLR9 activation in human pDCs but also confirm Choi and colleagues' observation [23] that TSG-6 induced a negative feedback loop to reduce the inflammatory response.

TSG-6 is known to be induced by agonists like lipopolysaccharide (LPS) and other inflammatory cytokines such as IL-1*β* and TNF-*α* on monocytes, macrophages, monocytes derived dendritic cells, polymorphonuclear leukocytes, and mesenchymal stem cells [35]. In addition to cytokines, growth factors like TGF-*β*1 can also induce TSG-6 expression in human smooth muscle cells. The induction of TSG-6 by cytokines and growth factors is tightly controlled by distinct pathways, and TSG-6 induction is unique in different cell types [14]. Our finding, for the first time, reveals that CpG-A and R837 can induce TSG-6 expression in human pDCs. It implies that the induction is not solely limited through LPS [35] via the TLR4 pathway, but TLR9 and TLR7 pathways are also involved.

Antigen presenting cells are the first type of cells recruited into tissues in response to foreign antigens or injury, and an uncontrolled inflammatory cytokine release would lead to adverse consequences such as tissue damage. Thus, having an immediate negative feedback response is essential. The induction of TSG-6 by TLR7 and TLR9 specific agonists CpG-A and R837 in GEN2.2 may provide evidence that TSG-6 is an important regulator to restore immune homeostasis in the cellular microenvironment. Augmented level of TSG-6 found in synovial fluids of patients with arthritis and the effective anti-inflammatory action of rhTSG-6 in vivo further suggest that TSG-6 may participate in a cytokine-initiated feedback loop which downregulates the inflammatory response [11, 15, 18, 36].

Continuous IFN-*α* and proinflammatory cytokine release is one of the most important disease mechanisms observed in SLE patients, and pDC is the major IFN-*α* producing cells in vivo [31]. Current treatment relies heavily on corticosteroids and other immune-suppressants to dampen the inflammation, which may be suboptimal in severe disease phenotypes. Moreover, these medications can increase risk of infections and have multiple drug related adverse effects. Thus, new treatment strategies are important to improve the prognosis of SLE with minimal therapy related complication. Advances in SLE treatment currently encompass the use of immunomodulators including prasterone (synthetic dehydroeipandrosterone), vitamin D, hydroxychloroquine, and belimumab. In particular, hydroxychloroquine, an antimalarial drug, has been shown to improve the prognosis and survival outcome of SLE patients [37–39]. Hydroxychloroquine, as shown by Sacre and colleagues, could reduce the capacity of pDCs in producing IFN-*α* and TNF-*α* upon in vitro stimulation with TLR9 and TLR7 agonists in SLE patients [31]. Similarly, in our study, we found that TSG-6 was able to downregulate IFN-*α* and TNF-*α* production in CpG-A or R847 stimulated human pDCs, making TSG-6 a potential immunomodulator for autoimmune disorders such as SLE.

Rapamycin has been used in clinical study for SLE patients who are refractory to first-line medications and was found to be effective in lowering disease activity and steroid dependence [40]. Several mechanistic studies evaluated the effect of rapamycin on blocking mammalian target of rapamycin (mTOR) activation [41–44]. Boor and colleagues found that rapamycin reduced IFN-*α* expression in TLR7 pathway more effectively than that of TLR9, while suppression of inflammatory cytokine was equally effective for both pathways [41]. A very similar phenomenon was seen in our study, in which we found that both TLR7 and TLR9 induction would result in different immunosuppressive dynamics of TSG-6 in downregulating IFN-*α* and TNF-*α*. Moreover, the intensity of TSG-6 expression induced by these two pathways also differed. TSG-6 was more effective in decreasing R837 induced IFN-*α* in human pDC and additionally, the suppressive effect was more evident on IFN-*α* than on TNF-*α* in both CpG-A or R837 activated human pDCs. We hypothesize that the suppressive effect of TSG-6 may be pathway-specific and that the downregulation seemed to be more prominent in TLR7 pathway compared with TLR9 pathway in human pDC. Furthermore, TSG-6 transcription was induced at a greater level in R837 stimulated human pDCs, although the overall protein expressions of TSG-6 in both CpG-A and R837 stimulated pDCs were similar. The difference in the dynamics of TSG-6 transcription in contrast with a comparable protein expression induced by CpG-A and R837 activation may suggest that the intermittent signalling molecules involved in TSG-6 induction are distinct in both TLR9 and TLR7 pathways. Overall, we found that TSG-6 could effectively downregulate both IFN-*α* and TNF-*α* in pDCs cell-line GEN2.2 and human peripheral blood pDCs following CpG-A and R837 stimulation.

To elucidate the possible signalling molecules involved, we performed real-time RT-PCR on GEN2.2 stimulated with CpG-A, with or without TSG-6, and found that IRF7, IFN-*α*2, and TNF-*α* gene expressions were decreased. IRF7 is an important regulator of type I IFN expression, and aberrant production of type I IFN is often associated with autoimmune disorders such as SLE. Stringent regulation of IRF7 expression and its activity is therefore very important in determining optimal type I IFN production for physiological functions. Posttranslational modifications such as serine phosphorylation are important in regulating IRF7 activation [45]. Our findings showed that TSG-6 reduced IRF7 phosphorylation in CpG-A or R837 stimulated GEN2.2. This functionality observation correlates with the decrease of TNF-*α* and IFN-*α*. A similar IRF7 negative regulatory mechanism was described by Liang and colleagues [46], in which they observed that ATF4, a stress response molecule induced by viral infection, is able to inhibit IRF7 activation by inhibiting the phosphorylation of Ser477/Ser479 by TBK1 and IKK*ε*, thereby suppressing the gene expression of IFN-*α* and IFN-*β*.

Choi et al. observed that the negative feedback of TSG-6 on resident murine macrophages was dependent on CD44 [23]. This led us to investigate if this would also be the case in human pDCs. First, we found that human pDCs pretreated with TSG-6 followed by CpG-A or R837 stimulation had a higher transcriptional level of CD44 and surface expression compared with no TSG-6 pretreatment. This finding suggested that the increased CD44 expression might be a response to TSG-6, and the cell surface expression of CD44 seen on untreated pDCs may suggest that CD44 might be preformed to be in a state ready to mount an immediate response. As described by Choi et al., CD44 was required for the inhibitory effect of TSG-6. We further showed that the effect of TSG-6 on human pDCs was dependent on CD44, since blocking with CD44 antibody partially abrogated the inhibitory effect of TSG-6 on CpG-A or R837 stimulated GEN2.2. These results were consistent with the observations by Choi et al. that the inhibitory effect of TSG-6 on TNF-*α* expression in zymosan-stimulated murine macrophages was negated by CD44 blocking antibody [23].

Although we have shown that TSG-6 exerted an anti-inflammatory effect that was CD44 dependent, we cannot negate the effect of TSG-6 on modulating hyaluronan (HA) interaction with CD44 [47, 48]. HA synthesized by dendritic cells mediates the interaction between T-cells that is important for antigen activation [49]. Lesley et al. suggested that binding of soluble TSG-6 and HA complexes to surface of leukocytes could inhibit interaction of circulating cells with endothelium, resulting in an anti-inflammatory effect [48]. Therefore, it was also possible that the CD44 dependent anti-inflammatory effect we observed (Figure 6) could have been a result of altered CD44 and HA interaction, since TSG-6 might prevent HA binding to CD44. However, in another study by Kawana et al. employing the zymosan-induced arthritis (ZIA) model in CD44 knockout mice, CD44 was required for suppression of inflammation. In particular, they showed that the inhibitory effect of low molecular weight-HA (3 and 22 kDa) for LPS-induced NF-*κ*B activation was not related to CD44 expression and that high molecular weight-HA (940 kDa) might not be involved in the inhibitory effect of CD44 on LPS-induced NF-*κ*Β activation [50]. Thus, it seems likely that TSG-6 may have two roles in suppressing inflammation: (1) TSG-6 assembly with HA would reduce recruitment of leukocytes and activation of T-cells; (2) TSG-6 interaction with CD44 may result in a direct suppression of inflammation.

Our observation, thus far, points towards a similar negative feedback mechanism, in which TSG-6 could be induced by TLR9 or TLR7 specific ligands and might be important in regulating the TLR9 and TLR7 pathways. Furthermore, exogenous addition of TSG-6 was able to downregulate CpG-A or R837 primed pDCs by decreasing IRF7 phosphorylation. Our data suggested that TSG-6 might play an immune-regulatory role in pDCs and provided us with a potential therapeutic strategy for SLE patients.

## 5. Conclusions

We have described, for the first time, a regulatory mechanism on TLR7 and TLR9 pathways in human pDCs exerted by TSG-6, which suppresses proinflammatory cytokines TNF-*α* and IFN-*α*, by decreasing the IRF7 phosphorylation.

## Supplementary Material

Supplementary Figure S1. GEN2.2 were incubated with different doses of TSG-6 (10-1000 ng/mL) over-night, followed by stimulation with CpG-A (2 uM) or R837 (10 ng/mL) for 6 hr. Supplementary Figure S2. Gating Strategy of human pDC from PBMC. Supplementary Table S1. Panel of flow-antibodies used for staining. Supplementary Table S2. Primer Sequence for Real-Time PCR
