# Supplementary material for: TSG-6 Downregulates IFN-Alpha and TNF-Alpha Expression by Suppressing IRF7 Phosphorylation in Human Plasmacytoid Dendritic Cells
Source: Mediators Inflamm. 2017 Mar 6;2017:7462945. doi: 10.1155/2017/7462945 (PMC5358455; doi:10.1155/2017/7462945)
Supplement: Supplementary file 1 — Supplementary Figure S1. GEN2.2 were incubated with different doses of TSG-6 (10-1000 ng/mL) over-night, followed by stimulation with CpG-A (2 uM) or R837 (10 ng/mL) for 6 hr. Supplementary Figure S2. Gating Strategy of human pDC from PBMC. Supplementary Table S1. Panel of flow-antibodies used for staining. Supplementary Table S2. Primer Sequence for Real-Time PCR [file 7462945.f1.docx]

**Supplemental Information**

**TSG-6 down regulates Interferon-alpha and TNF-alpha expression by suppressing IRF7 phosphorylation activity in human plasmacytoid dendritic cells**

*Kui L, Chan GC, Lee PPW*

**Supplemental Inventory**

**Supplemental Figures**

Figure S1, Related to Figure 1

Figure S2, Related to Materials and Methods (Flow cytometry)

Table S1, Related to Materials and Methods (Flow cytometry)

Table S2, Related to Materials and Methods (RNA isolation and quantitative real-time PCR)


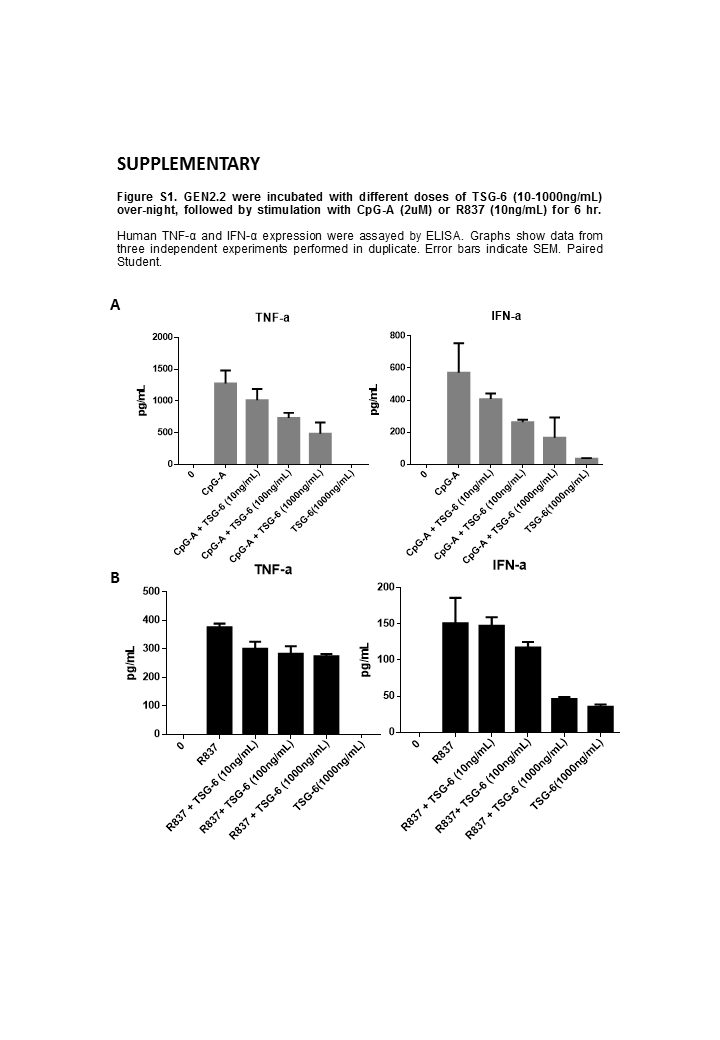


**Figure S2. Gating Strategy of human pDC from PBMC**

**A**


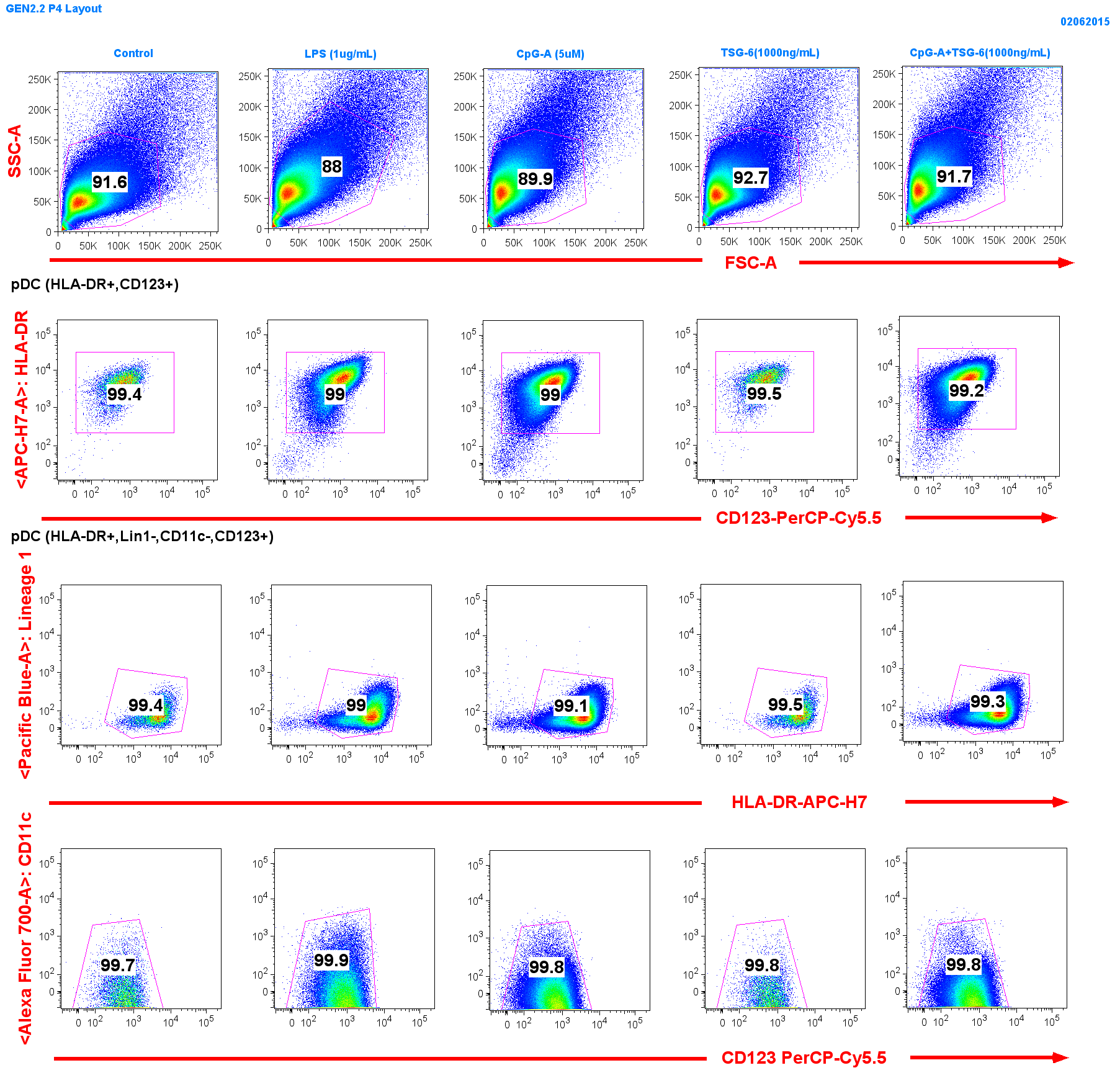


**SSC-A**

**FSC-A**


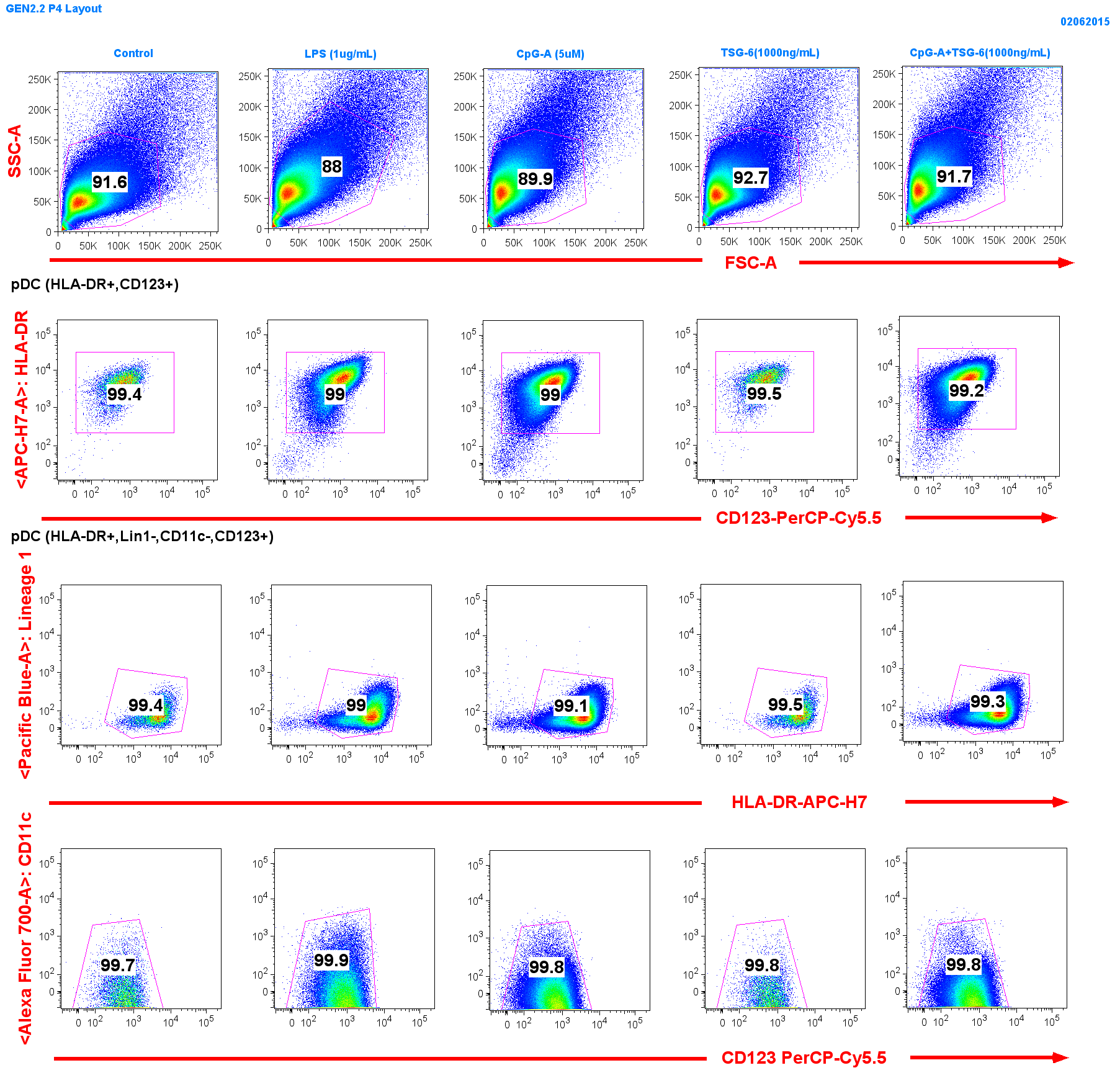


**Lineage 1- PacificBlue**

**HLA-DR APC-H7**


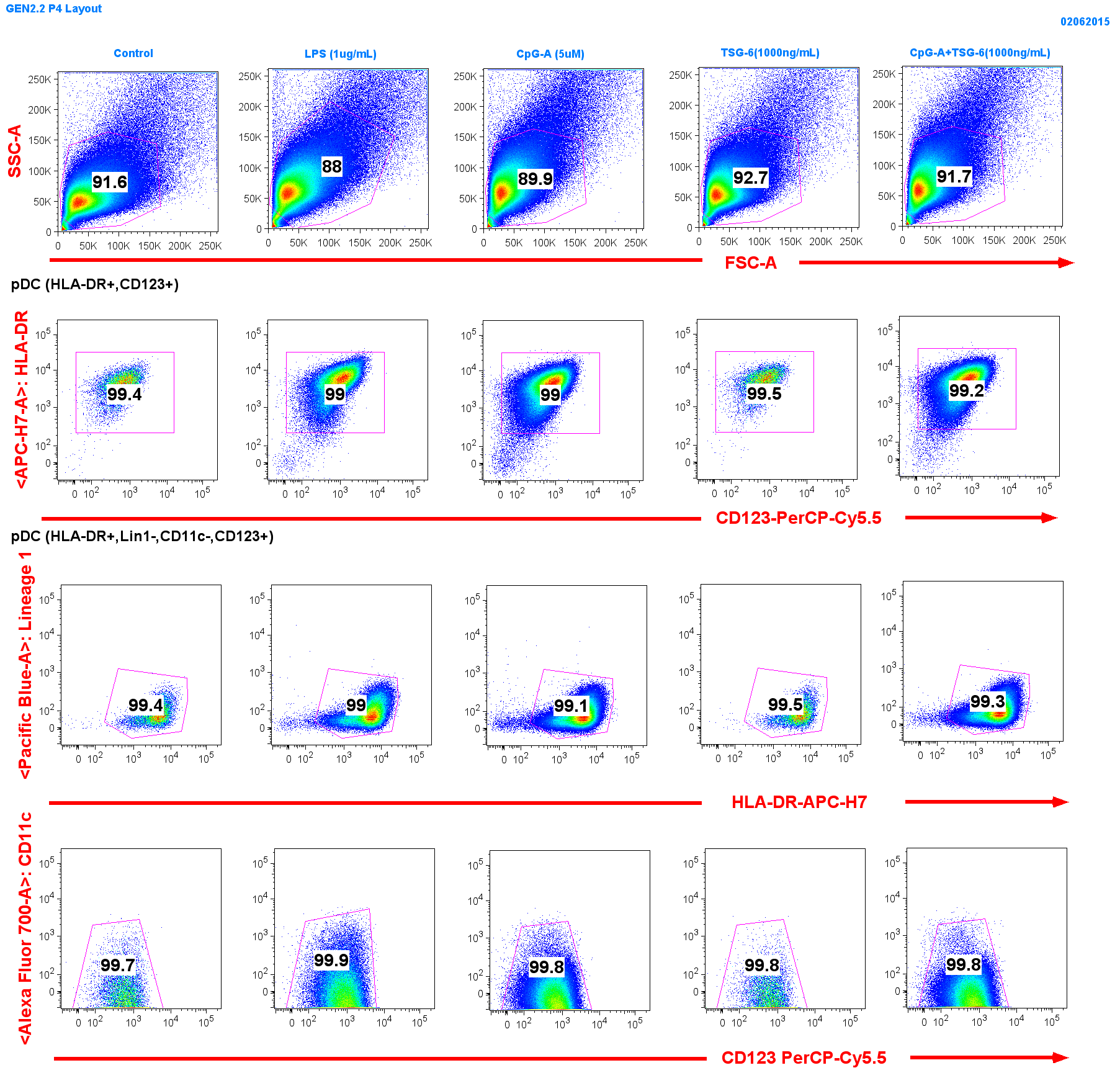


**CD123-PerCP Cy5.5**

**CD11c AlexaFluor 700**

GEN 2.2 pDC cell-line is defined as HLA-DR^+^ Lineage-1^-^ CD11c^-^ CD123^+^ cells, as indicated in the gating strategy (A).

**B**

pDC population were gated from the PBMC and defined as HLA-DR^+^ Lineage-1^-^ CD11c^-^ CD123^+^ cells, as indicated in the gating strategy (B).

**Table S1. Panel of flow-antibodies used for staining**

| **Parameter** | | **Manufacturer** | **Cat. No.** |
| --- | --- | --- | --- |
| Pacific Blue | Lineage 1  (Lineage Cocktail CD3/14/16/19/20/56) | BD Biosciences | 348805 |
| PerCP Cy5.5 | CD123 | BD Biosciences | 560904 |
| AlexaFluor 700 | CD11c | BD Biosciences | 561352 |
| APC H7 | HLA-DR | BD Biosciences | 561358 |
| PE | IL-10 | BD Biosciences | 559337 |
| FITC | TNF-a | BD Biosciences | 554512 |
| APC | IFN-a | Miltenyi Biotec | 130-099-214 |
| ALEXA647 | IRF-7(PS477/PS479) | BD Biosciences | 558630 |
| PE | CD44 | BioLegend | 338808 |
| PE | IRF-7 | BD Biosciences | 558706 |

**Table S2. Primer Sequence for Real-Time PCR:**

| hTSG-6 (TNFAIP6) | Forward: | 5'-TTTCTCTTGCTATGGGAAGACAC-3' |
| --- | --- | --- |
|  | Reverse: | 5'-GAGCTTGTATTTGCCAGACCG-3' |
| hB-actin | Forward: | 5’-AGAGCTACGAGCTGCCTGAC-3’ |
|  | Reverse: | 5’-AGCACTGTGTTGGCGTACAG-3’ |
| hIRF-7 | Forward: | 5'-TGGTCCTGGTGAAGCTGGAA-3' |
|  | Reverse: | 5'-GATGTCGTCATAGAGGCTGTTGG-3' |
|  |  |  |
| hIFN-a2    hTNF-a | Forward: | 5'-GCTTGGGATGAGACCCTCCTA-3' |
|  | Reverse: | 5'-CCCACCCCCTGTATCACAC-3' |
| hCD44 | Forward: | 5'-CCCAGGGACCTCTCTCTAATCA-3' |
|  | Reverse: | 5'-AGCTGCCCCTCAGCTTGAG-3' |
|  | Forward: | 5'-CTGCCGCTTTGCAGGTGTA-3' |
|  | Reverse: | 5'-CATTGTGGGCAAGGTGCTATT-3' |
